# Assessing Drug–Drug Interaction and Food Effect for BCS Class 2 Compound BI 730357 (Retinoic Acid-Related Orphan Receptor Gamma Antagonist, Bevurogant) Using a Physiology-Based Pharmacokinetics Modeling (PBPK) Approach with Semi-Mechanistic Absorption

**DOI:** 10.3390/pharmaceutics17030314

**Published:** 2025-03-01

**Authors:** Tobias Kanacher, Erik Sjögren, Julia Korell, Elodie L. Plan, Jose David Gómez-Mantilla, Ibrahim Ince

**Affiliations:** 1 Pharmetheus AB, 753 19 Uppsala, Sweden; 2 Boehringer Ingelheim Pharmaceuticals, Inc., Ridgefield, CT 06877, USA; 3 Boehringer Ingelheim Pharma GmbH & Co. KG, 55218 Ingelheim, Germany

**Keywords:** BI 730357, bevurogant, DDI, drug–drug interaction, PBPK, pharmacokinetics, physiologically based pharmacokinetic modeling, retinoic acid-related orphan receptor gamma inhibitor, RORgamma inhibitor, simulation

## Abstract

**Background**: The drug candidate BI 730357 is a Biopharmaceutics Classification System (BCS) Class II compound showing atypical absorption after oral administration in fasted and fed healthy individuals, for which conventional traditional deconvolution methods could not explain formulation dependencies. **Methods**: A physiologically based pharmacokinetic (PBPK) model of BI 730357 was developed using the Open Systems Pharmacology (OSP) PBPK software tool PK-Sim^®^, which could describe the pharmacokinetics in fasted and fed subjects after single and multiple doses. A Weibull function was used to describe the in vivo formulation kinetics, whereas colonic absorption was adopted as the main driver to describe the late phases of observed pharmacokinetics after oral administration. The food effect was applied using the implemented feature PK-Sim^®^. **Results**: The model accurately predicted an observed itraconazole drug–drug interaction (DDI) in fasted subjects and was used to explore the effects of the strong CYP3A4 inducer rifampicin on the pharmacokinetics of BI 730357 after administration in fed subjects. **Conclusions**: The combined results suggest that the BI 730357 PBPK model with semi-mechanistic absorption can prospectively explore the effects of CYP3A4 inhibitors and inducers on the pharmacokinetics after administration in fed or fasted subjects within the given dose range.

## 1. Introduction

**Figure 1:**
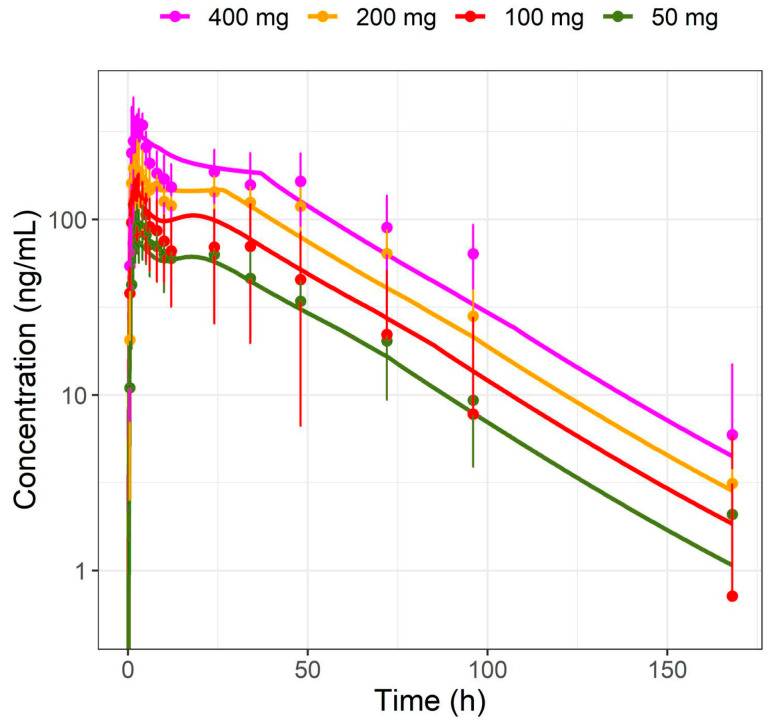
Observed and simulated plasma concentration–time profiles in fasted subjects over a 50 to 400 mg tablet single dose range in study 1407-0001. Dots represent the observed medians with standard deviations, and lines represent simulated values using parameters of a typical study individual.

The drug candidate BI 730357 is a competitive antagonist to the retinoic acid-related orphan receptor γ and is investigated as an oral treatment of plaque psoriasis. BI 730357 is a lipophilic base and a Biopharmaceutics Classification System (BCS) Class II compound [1,2,3,4] showing high permeability and pH-dependent low solubility. In the first-in-human single ascending dose study, sub-proportionality in dose-exposure was observed for doses above 50 mg (Figure S1). A late-phase absorption peak was observed in the multiple ascending dose study and appeared to be more pronounced at higher doses (Figure 1), which was also investigated in an accompanying BI 730357 population PK analysis [5].

At the 50 mg dose, the absolute oral bioavailability was ~70%. For 25 mg to 100 mg doses, the mean half-life ranged from 22.5 to 29.8 h. Ninety percent of BI 730357 hepatic metabolism was attributed to CYP3A4 based on human hepatocyte experiments, which was consistent with CYP reaction phenotyping studies [6]. In initial clinical food effect studies, positive food effects were observed at doses above 50 mg. BI 730357 exposure data also implied formulation dependencies and a high interstudy variability that could not be explained by typical interindividual variability in biometrics or physiology. Additionally, an adequate in vitro in vivo correlation (IVIVC) among the different formulations could not be established by traditional deconvolution methods.

Physiologically based pharmacokinetic (PBPK) modeling has emerged as an important tool in drug development, allowing the prediction of drug–drug interactions (DDI), investigation of food effects, and rational formulation development [7,8]. Recent guidelines from the US FDA [9] and the European Medicines Agency [10] advocate for using PBPK models to assess DDI risks in new investigational drugs. These models replicate how drugs move through the body by dividing it into compartments connected by blood flow, mirroring the circulatory system. PBPK models distinguish between drug-specific parameters (like molecular weight and lipophilicity) and biological system-specific parameters (such as organ sizes and blood flow rates), making them versatile for different populations and enabling drug substitution by adjusting these parameters. The mechanistic nature of PBPK models enhances the understanding of physiological processes that influence drug pharmacokinetics [11,12].

This method has become a key component of drug development, with ongoing improvements to ensure the quality and reliability of specialized PBPK software platforms like PK-Sim^®^. The Open Systems Pharmacology (OSP) PBPK tool PK-Sim^®^ uses reusable building blocks categorized into individuals, populations, compounds, formulations, administration protocols, events (such as food intake), and observed data to construct models. These building blocks can be combined and modified to suit various scenarios.

In this work a PBPK modeling case using PK-Sim^®^ (version 9.1) is presented. The aims of this analysis were to (1) investigate the non-linearity in the PK of BI 730357 using the PBPK modeling approach, describing the absorption, distribution, metabolism, and excretion (ADME) characteristics after oral administration with and without food intake, and (2) explore any potential DDI towards CYP3A4 inhibitors and inducers through simulations with BI 730357 as a victim, in the absence of a clear understanding of the in vivo formulation kinetics.

## 2. Materials and Methods

**Figure 2:**
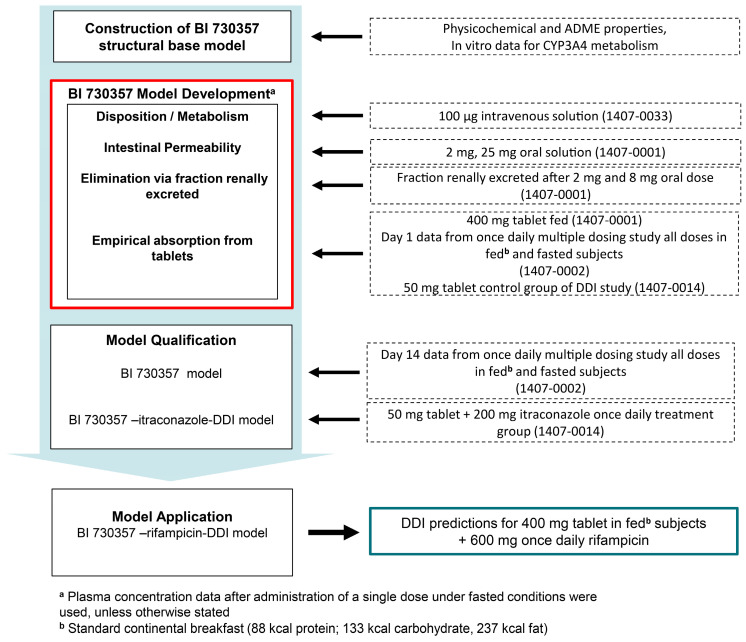
Workflow of model building, evaluation, and application steps.

The model development steps and the clinical data used are illustrated in Figure 2 and detailed in the following sections.

### 2.1. Data Collection

Input data used included experimentally measured in vitro parameters (Table 1). Drug concentration measurements from 5 Phase I studies in healthy male subjects were used for the model building, model evaluation, and model application steps. The study protocols were approved by the institutional review board or ethics committee at each study site, and the studies were conducted in accordance with Good Clinical Practice guidelines and the ethical standards laid down in the Declaration of Helsinki. All subjects provided written informed consent before any study procedures were undertaken. The clinical data, including study number, number of subjects, subject feeding state, drug formulation, dosing regimen, and measurement matrix, are summarized in Table S1. BI 730357 was administered orally in the 5 studies, using an oral solution and two immediate release tablet formulations with different milling grades. In study 1407-0033, an intravenous (IV) microtracer dose of BI 730357 (C-14) was administered 15 min after a single oral dose to investigate absolute oral bioavailability.

### 2.2. Model Development

The fundamental concepts of PBPK modeling have been described in detail by Kuefer et al. [12]. As a first step, the BI 730357 structural base PBPK model was developed based on physicochemical data, in vitro data, and pre-clinical pharmacokinetics observations (Figure 2 top panel).

A stepwise approach was used to further inform disposition (i.e., distribution and elimination) in the PBPK model of BI 730357 using clinical plasma concentration–time profiles after intravenous (IV) administration (study 1407-0033) (Figure 2 middle panel). The elimination of the compound was structurally implemented via CYP3A4 metabolism as a first-order intrinsic clearance process. The relative CYP3A4 expression in the different organs is based on high-sensitive real-time RT-PCR [13]. Absolute tissue-specific expressions were obtained by considering the respective absolute concentration in the liver. The PK-Sim^®^ database provides a default value of CYP3A4 4.32 µmol/L in the liver [14] and assumes 40 mg protein per gram liver. Different distribution models in PK-Sim^®^ were tested, and for the prediction of the tissue:plasma partition coefficient, the Rodgers and Rowland model provided the best fit. In the next step, clinical plasma concentration–time profile data from oral solution administrations of a single rising dose study (1407-0001) were used to evaluate the pre-systemic clearance, transcellular intestinal permeability, and solubility of the solution formulation.

Further, renal clearance that was initially implemented by glomerular filtration to only account for passive processes was informed by experimental data on the fraction renally excreted (study 1407-0001).

Finally, the dissolution kinetics of the used tablet formulation were estimated using an empirical Weibull dissolution function that was fitted to plasma–concentration time profiles of single or the first-day multiple-dose tablet data (studies 1407-0001, 1407-0002, and 1407-0014).

For model qualification, plasma-concentration time profiles from day 14 of the multiple dose study (1407-0002, Figure 2 middle panel) and the treatment group (50 mg BI 730357 tablet + 200 mg itraconazole once daily treatment) from the DDI study (1407-0014) were used to verify the assumed fraction metabolized by CYP3A4.

To predict DDI with rifampicin as a perpetrator, a PBPK model of rifampicin acting as a strong CYP3A4 inducer, administered 600 mg once daily, was coupled with the BI 730357 PBPK model. Here the empirical absorption, fitted to data from two dose groups from study 1407-0001 and 1407-0002, where 400 mg BI 730357 was administered to fed subjects, was used to predict the impact on BI 730357 pharmacokinetics after administration to fed subjects (Figure 2 bottom panel).

### 2.3. Simulations

Simulations were run for a standard European male individual set to PK-Sim^®^ (Version 9.1) default biometrics. The created virtual individuals were based on appropriate populations incorporated in PK-Sim^®^ [10]. Enzyme and transporter expression levels were obtained from the PK-Sim^®^ gene expression database [15,16], and the demographic variables were taken from clinical study data. Food effect was incorporated as a built-in feature in PK-Sim^®^. Population simulations are performed within PK-Sim^®^ by using a population of virtual individuals. The characteristics of such a population are defined by race, proportion of males/females, age range, and range of body weight, height, and body mass index. From these data, the parameters relevant for PBPK modeling, such as organ volumes and blood flows, are generated for each virtual individual. The algorithm to generate a population also considers inter-individual variability and employs a Monte Carlo method to randomly vary system parameters according to the distributions available within the population’s databases [10].

Dynamic mechanistic models for modulating effects on enzyme and transporter activity are available within PK-Sim^®^ and can be added to the simulations [17]. For competitive enzyme inhibition, the unbound concentration of the inhibitor at the site of the enzyme (I) and the in vitro inhibition constant (K_i_) are driving the interaction. The intrinsic clearance (CL_int_) in the presence of a competitive inhibitor, apparent intrinsic clearance (CL_int,app_), is represented by the equation:

CL_int,app_ = CL_int_ × (1 + [I]/K_i_)

For enzyme induction, the unbound concentration of the inducer at the site of the enzyme (I) as well as the induction parameters, maximum effect (E_max_) and concentration at half maximum effect (EC_50_), are driving the interaction by increasing the enzyme synthesis rate (R_syn_). E_max_ is the maximal induction effect in vivo, and EC_50_ is the concentration to reach half of E_max_. The enzyme turnover is affected according to the following equations:

d[E]/dt = R_syn,app_ − k_deg_ × [E]

R_syn,app_ = R_syn_ × (1 + (E_max_ × [I])/(EC_50_ + [I]))

### 2.4. Parameter Estimation

The model development process included parameter estimation to refine model performance by optimization of the key parameter values. The “Parameter Identification” functionality included in PK-Sim^®^ was used. This is a statistical optimization functionality where the residuals between observed data and corresponding simulation output were minimized by varying selected input parameters in each range. Multiple optimizations with randomized initial values using the Levenberg–Marquardt algorithms or Monte Carlo simulations were applied.

### 2.5. Model Evaluation

Model performance was assessed primarily by comparing simulated to observed data (concentration–time profiles and summary PK parameters), including uncertainties, such as a mean with a 90% prediction interval. For the concentration–time profiles, the comparison was made both on the absolute concentrations and on the shape of the profiles.

The simulations were based on a representative typical individual or a virtual population of 100 individuals to evaluate the appropriateness of the models, depending on the stage of the analysis. The demographics of the virtual populations were set to match the clinical study populations. The predictive performance of PK parameters and drug–drug interaction ratios was assessed based on a predefined criterion of a 1.5-fold range of the observed/simulated ratio. The assessed PK parameters were the area under the concentration–time curve (AUC) and peak plasma concentration (C_max_). Population geometric mean of the concentration–time profiles and PK parameters were predicted and compared to observed data.

### 2.6. Additional Models

The previously developed itraconazole and rifampicin PBPK models and their evaluation reports are available at the Open Systems Pharmacology GitHub repository [18,19] along with information on the qualification of the models and interaction networks [5]. The model versions used were tagged v9.1.1 and committed on 14 December 2020. The itraconazole model has been qualified for investigating the impact of strong CYP3A4 inhibitors on CYP3A4 victim substrates [18], and the rifampicin model has been qualified for investigating the impact of strong CYP3A4 inducers on CYP3A4 victim substrates [19].

### 2.7. Data Analysis

The analyses were performed with the PK-Sim^®^ and MoBi^®^ components of the Open Systems Pharmacology Suite [20] version 9.1. Simulations were performed in a virtual healthy population called “European P pg modified, CYP3A4, 36 h, EHC” in PK-Sim^®^, and dosing regimens and demographics from the respective clinical studies were applied.

This virtual healthy population is based on a typical whole-body model in PK-Sim^®^, which includes 15 organs or tissues connected by the circulating blood system and defined by tissue volume, composition, and blood flow. For small molecule simulations, each organ consists of four compartments: plasma, red blood cells, extracellular space, and intracellular space [21]. The absorption model in PK-Sim^®^ divides the gastrointestinal tract into 12 segments, defined by sub-tissue volume, transit time, and pH. Each of the segments consists of lumen, mucosal tissue, and non-mucosal tissue. For the simulation of oral solution formulations, the lumen of each segment is further divided into two compartments, representing the drug in solution and the fluid volume available in the luminal segment [22]. For oral solid formulation simulations, a compartment of the solid formulation is also added, which is connected to the dissolved drug [23].

## 3. Results

### 3.1. Data Exploration

**Figure 3:**
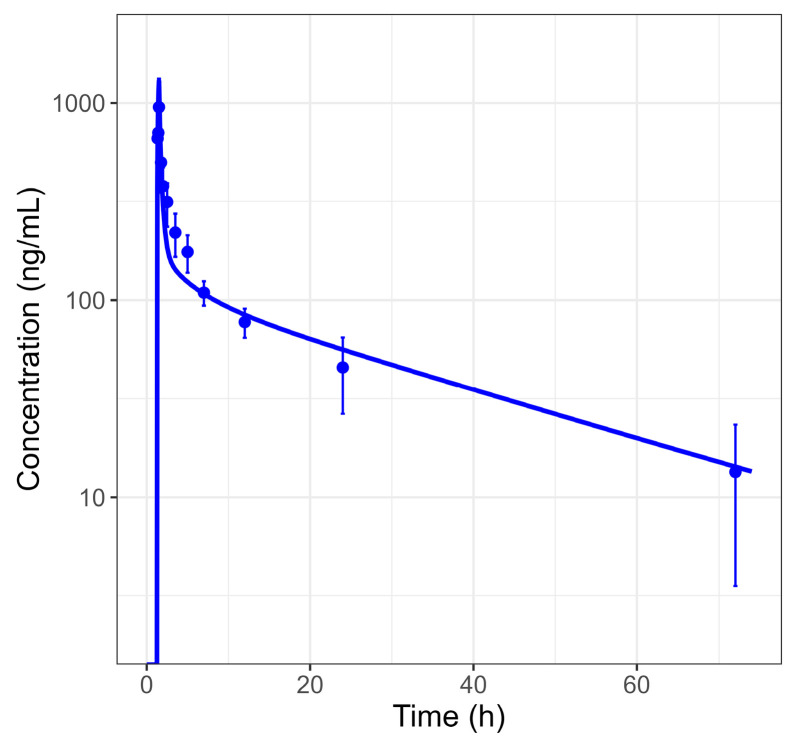
Observed and simulated plasma concentration–time profile in fasted subjects after 100 μg microtracer dose (normalized to a 35.26 mg dose for comparison with other doses) as intravenous solution administration in study 1407-0033. Dots represent the observed medians with standard deviations, and the line represents simulated values using parameters of a typical study individual.

**Figure 4:**
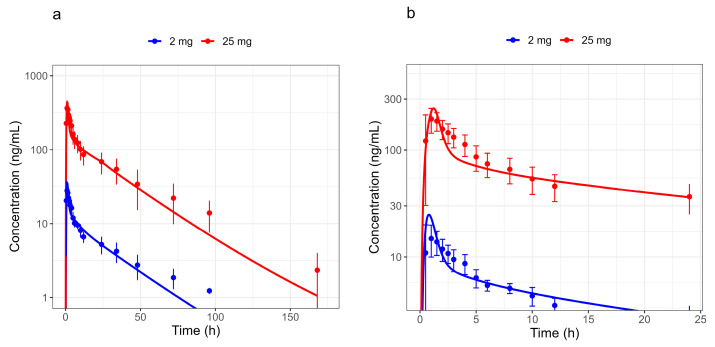
Observed and simulated plasma concentration–time profiles in fasted subjects after 2 mg and 25 mg oral solution administration in study 1407-0001. Plotted on a semi-log scale (**a**) shows full timescale and (**b**) focusses on the initial 24 h. Dots represent the observed medians with standard deviations, and lines represent simulated values using parameters of a typical study individual.

Plasma concentration–time profiles and urinary fractions excreted of BI 730358 from clinical studies were explored (Figure S1) in an accompanying BI 730357 population PK analysis [24]. Absorption from oral solid forms appeared to be bi-phasic with the first peak around 2 to 4 h and the second peak between 16 and 36 h after the last dose (Figure 1). Since the late absorption was not timely related to food intake and not present in profiles after IV (Figure 3) or solution (Figure 4) administration, it was assumed not to be related to enterohepatic circulation.

A dose effect (dose-exposure sub-proportionality) was observed in subjects both in the fasted and the fed state, where higher daily doses were associated with lower bioavailability. A positive food effect on bioavailability was noted, where administration in the fed state generally displayed a longer absorption phase with higher bioavailability and later C_max_ compared with the fasted state. In addition, the range of dose proportionality in exposure seemed larger in the fed state (50 to 200 mg) compared to the fasted state (25 to 100 mg).

The slope of plasma concentration versus time after the last dose appeared to be similar among the different doses after multiple dosing, which supports the assumption of linear clearance. A comparison of concentration–time profiles of subjects administered the same dose and formulation in different studies indicated large interindividual and interstudy variability.

### 3.2. Physiologically Based Pharmacokinetic Model

The model described observations after BI 730357 IV administration well (Figure 3), and the parameters of the final model are presented in Table 1. Based on numerical and visual diagnostics, the Rodgers and Rowland model [25,26,27] best described the disposition of BI 730357, in combination with the estimated lipophilicity, intestinal permeability, and clearance parameters in Table 1. The model underestimated in vivo permeability in comparison to the in vitro value, which is most likely due to the low in vivo absorption and the difficulty of discriminating between solubility and permeability effects on absorption [28]. A major part of the clearance was attributed to a first-order intrinsic CYP3A4 clearance process. Supported by observations, it was found that renal elimination only accounted for a minor part of total clearance and was assumed to be driven by active reabsorption in the proximal tubules of the kidney. No drug transporters were included in the final model, as in vitro data suggested that BI 730357 is not a substrate for P-glycoprotein, breast cancer resistance protein, or organic anion transporting polypeptides 1B1 and 1B3 transporters.

### 3.3. Description of Absorption

**Figure 5:**
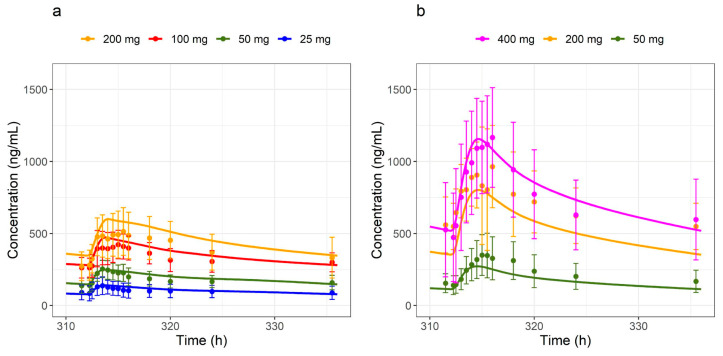
Observed and simulated plasma concentration–time profiles after once daily administration of 25 mg to 200 mg in the fasted state (**a**) and 50 mg to 400 mg in the fed state (**b**) for 14 days in study 1407-0002. Dots represent the observed medians with standard deviations, and lines represent simulated values using parameters of a typical study individual.

BI 730357 displayed dose-exposure sub-proportionality that was described empirically by adopting a solid formulation with Weibull release and the default multicompartmental transit and absorption model in PK-Sim^®^ and adequately predicted the pharmacokinetics of BI 730357 in study 1407-0001 (Figure 1) over the entire time course. By fitting Day 1 data with the empirical absorption model and extrapolating to Day 14, we were able to predict study 1407-0002 steady-state observations in the fasted state for the 25 mg to 200 mg dose range and in the fed state for the 50 mg to 400 mg dose range (Figure 5), without the need for further refining the model. The fitting parameters for the empirical Weibull dissolution function are listed in Table 2.

### 3.4. Prediction of Itraconazole Drug–Drug Interaction

**Figure 6:**
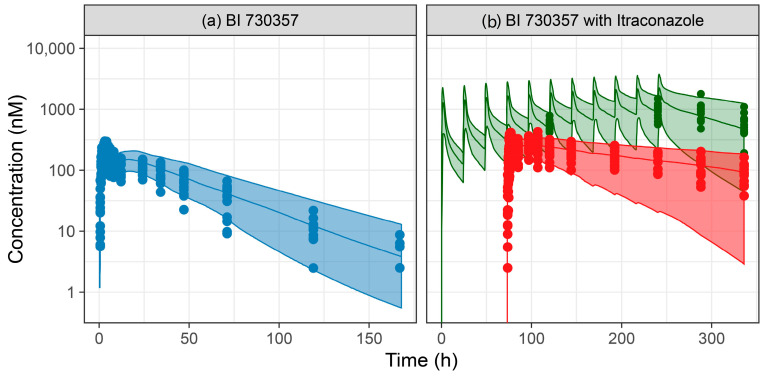
Observed and simulated plasma concentration–time profiles in fasted subjects in the drug–drug interaction study 1407-0014. Dots represent individual observations, and lines and shaded areas represent the simulated geometric mean with the 5–95% percentile range of 100 virtual individuals. (**a**) Control group (blue) single dose BI 730357 50 mg tablet (**b**) Treatment group (red) single dose BI 730357 50 mg tablet with 200 mg itraconazole (green) once daily from Day −3.

To predict itraconazole DDI, the empirical Weibull dissolution function was fitted to observations after oral administration of 50 mg BI 730357 in study 1407-0014 (Table 3). The PBPK model for itraconazole adequately described the effects of inhibition of CYP3A4 metabolism on BI 730357 pharmacokinetics as a victim drug when the itraconazole model template, including its 4 metabolites [29] and inhibition constants described in the literature, was applied as a perpetrator (Figure 6). The simulations slightly underpredicted the area under the plasma concentration curve (AUC) ratios and maximum plasma concentration (C_max_) ratios for the 50 mg dose due to a minor overprediction of the exposure in the control group (Table 4). However, the predicted AUC and C_max_ geometric means were within the limits of the 5% and 95% percentiles of the observations.

### 3.5. Prediction of Rifampicin Drug–Drug Interaction

**Figure 7:**
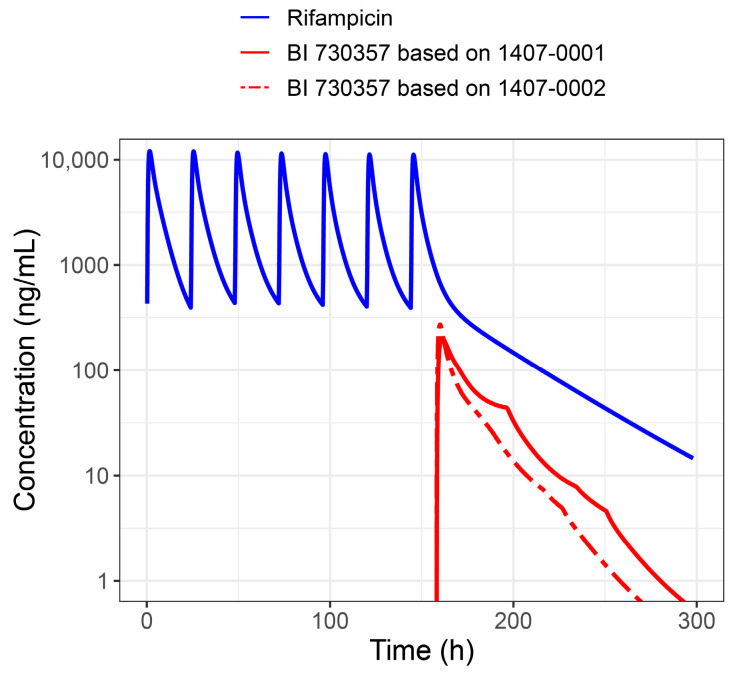
Prediction of drug–drug interaction after 600 mg rifampicin once daily and a BI 730357 400 mg single tablet dose in fed individuals. Empirical absorption in studies 1407-0001 and 1407-0002 was used to simulate BI 730357 concentrations in typical individuals.

To predict rifampicin (perpetrator) DDI with BI 730357 (victim), the empirical Weibull dissolution function was fitted to observations after oral administration of a 400 mg single dose in the fed state. Observations for BI 730357 were available from studies 1407-0001 and 1407-0002 (Table S1). The parameters derived after fitting the empirical Weibull dissolution function to observations from the two studies are available in Table 3, and the predicted rifampicin DDI is depicted in Figure 7.

The prediction based on the empirical absorptions from the two studies indicated incomplete absorption of BI 730357. The estimated dissolution shape parameter was 0.52 for study 1407-0001 and 0.16 for study 1407-0002. When the simulations using empirical absorption from the two studies were compared, the AUC and C_max_ ratios were similar (Table 5). The differences in AUC and C_max_ could reflect the extent of fraction absorbed from the two tablet formulations used, as well as expected interstudy variability.

## 4. Discussion

We developed a PBPK model describing the ADME characteristics of BI 730357 after oral administration with and without food using an empirical dissolution function and assuming a late intestinal absorption. Clinical study observations indicated a non-linear complex absorption and a dissolution characterized by a second late-phase absorption peak after tablet administration. Non-linearity during absorption can be due to Michaelis–Menten kinetics or saturable solubility. However, both would not explain the late phase absorption peak. An accompanying BI 730357 population PK analysis [24] could explain this sub-proportionality using a model with dual absorption paths and a first-order elimination. In the population PK model, most of the dose was absorbed via a sequential zero-order and first-order process after a brief absorption lag, and the remaining fraction of the dose was absorbed via a zero-order process after a long absorption lag.

For the PBPK model, an empirical fit of a dissolution function with an assumed elevated colonic absorption could accurately describe the observations across the full dose range. Using an empirical fit results in a model unable to describe non-tested doses within or outside the tested dose range. The empirical model must be fitted for each dose, as the time to reach 50% dissolution is dose dependent (Table 2), and therefore we cannot extrapolate to untested doses.

Although the AUC and C_max_ ratios of the simulated oral solution administration were within the generally accepted 1.5-fold evaluation limit in the PBPK modeling range (Figure 4a and Table 6), it did not optimally describe the clinical observations (Figure 4b). The observations after oral solution administration showed lower maximum plasma concentrations and a less steep decline immediately after T_max_ than expected from simulations using a solution formulation. This is indicating a lower solubility in the stomach and a subsequently reduced and delayed absorption from the small intestine than predicted by the model based on in vitro solubility. Several approaches were tested without success to describe the absorption after tablet administration, including pH dependency, particle size estimated from in vitro particle distribution measurements, and the supersaturation option available in the PK-Sim^®^ software.An approach that adequately described the observations was to assume a longer gastric emptying time, but the biological evidence was missing. Additionally, a longer gastric emptying time would have impacted the PK of the co-medication during drug–drug interaction predictions, which was not evident in the clinical observations in the drug–drug interaction study 1407-0014.

The late phase absorption peak between 16 to 36 h was prominent in the clinical observations after tablet administration (Figure S1). The late absorption peak was not present after the administration of IV and oral solutions and was unrelated to the time of food intake, ruling out enterohepatic recirculation. A delayed absorption due to active transportation was unlikely, as BI 730357 was not found to be a substrate of the most common transporters, such as P-glycoprotein and breast cancer resistance protein.

The challenges to describe the complex absorption behavior of BI 730357 were likely due to the complex chemical, physiological, and biochemical processes that occur from administration to absorption of BCS Class II compounds, which are not fully understood [28]. Further, the supersaturation model available in the PK-Sim^®^ software may have been more adequate if accounting for the complex dissolution, precipitation, and re-dissolution processes that have also been shown for other BCS Class II compounds [28,30].

Examples of how to describe late-phase absorption of similar low-soluble BCS Class II compounds have been presented [31,32,33]. In our analysis, based on the given compound properties, the assumption of colonic absorption [31] was considered a likely explanation and an adequate simplification. Potentially, continuous precipitation-dissolving processes while the compound moves through different parts of the intestine could also contribute to the delayed absorption, although it is difficult to differentiate from colonic absorption with the current model settings. Another possible explanation could be strong partitioning into colloidal structures, reducing the free active concentration in the small intestine and release in the jejunum or ileum at bile salt absorption [28,34].

The assumption of colonic absorption could be tested in the PBPK model since the colon in the gastrointestinal model implemented in the PK-Sim^®^ software is divided into six sub-compartments [18,19] and the model was well suited to test the hypothesis of a late colonic absorption. The use of the Weibull dissolution function together with an elevated solubility in the sigmoid colon was identified as an appropriate description of the late phase absorption. However, one must keep in mind that using the Weibull dissolution function assumes a dissolution profile independent of gastric emptying, gastrointestinal transit time, and pH effect. This might lead to limitations for specific applications and possibly to an underprediction of the interindividual variability related to these intestinal processes.

A more detailed investigation of the correlation between the predicted particle dissolution distribution and the in vitro dissolution profiles [35] was hampered by the high interindividual and interstudy variabilities. Therefore, the absorption issues were not fully elucidated, and no in vitro–in vivo correlation or safe space analysis was established. As the second objective was to explore drug–drug interaction, the use of estimated Weibull profiles to simulate in vivo release was deemed fit for purpose.

The large interindividual and interstudy variability in clinical study observations could not be fully explained by the PBPK model. Still, the model adequately described the data from all tablet single-dose groups (25 mg to 400 mg) in a fasted and fed state in study 1407-0002 (Figure 5). The observed versus simulated AUC and C_max_ ratios were close to 1 and within the generally accepted 1.5-fold evaluation limit in PBPK modeling (Table 7). The drug–drug interaction with itraconazole was successfully characterized by the model using empirical absorption scenarios, as the predicted geometric mean AUC and C_max_ values were well within the 5 to 95 percentiles around the observed geometric mean values (Table 4), suggesting that the fraction metabolized was well described. The pragmatic approach using the empirical Weibull function and the elevated colonic solubility in the multi-compartment GI model was also mirrored in the absorption assumptions in an accompanying BI 730357 population PKPD analysis [24]. That emphasized the utility of the approach and highlights the benefits when a PBPK and population PKPD model are used in parallel for investigating new chemical entities. As a next step, a retrospective evaluation should be performed to evaluate the predictive potential of the model for assessing the DDI of CYP3A4 perpetrators on BI 730357 PK.

## 5. Conclusions

We have developed a PBPK model of BI 730357 using the Open Systems Pharmacology (OSP) PBPK software tool PK-Sim^®^, which could describe the pharmacokinetics in fasted and fed subjects after single and multiple doses, using a Weibull function to describe the in vivo formulation kinetics, whereas colonic absorption was adopted as the main driver to describe the late phases of observed pharmacokinetics after oral administration. The model accurately predicted an observed itraconazole DDI in fasted subjects and was used to explore the effects of the strong CYP3A4 inducer rifampicin on the pharmacokinetics of BI 730357 after administration in fed subjects. We showed that estimation of empirical functions towards appropriate clinical data can overcome gaps in mechanistic knowledge and allow for prediction of different scenarios among the tested doses. The PBPK model using simple dissolution functions based on empirical fits could be further used in DDI assessment for the effect of CYP3A4 perpetrators on BI 730357 PK with and without food intake.

## Figures and Tables

**Table 1:** Initial and final values of the model parameters.

Parameter (Unit)	Initial Value	Final Value
Molecular weight ^a^ (g/mol)	532.62	532.62
pK_a_ ^a^	2.2 (base)	2.2 (base)
Log P	3.4 ^a^	2.8 ^b^
Intestinal permeability (10^−6^ cm/s)	110 ^c^	0.442 ^d^
Solubility ^e^ fasted state (mg/L)	19.45	19.45
Solubility ^f^ fed state (mg/L)	57.9	57.9
Colon transversum solubility fasted state (mg/L)	19.46 ^g^	637 ^d^
Fraction unbound in plasma ^a^ (%)	15.4	15.4
Hepatic CL_int_ (mL/min)	63.9 ^h^	154.71 ^b^
GFR fraction	1 ^i^	0.059 ^d^

^a^ Experimentally measured. ^b^ Optimized using data from study 1407-0033. ^c^ Caco-2 cell system. ^d^ Optimized using data from study 1407-0001. ^e^ FaSSIF-24 h pH 6.5. Used as reference solubility in PK-Sim^®^ to calculate pH-dependent solubility in the gastrointestinal tract. ^f^ FeSSIF-24 h pH 5.0. Used as reference solubility in PK-Sim^®^ to calculate pH-dependent solubility in the gastrointestinal tract. ^g^ Calculated in PK-Sim^®^ using reference solubilities in (e) according to the solubility at the pH of the colon transversum. ^h^ CYP3A4 metabolism. ^i^ PK-Sim^®^ parameter to describe renal clearance. Value 1 assumes that renal clearance is governed by passive glomerular filtration only. The value was adjusted to the measured mean fraction dose excreted. Abbreviations: CL_int_, intrinsic clearance; FaSSIF, fasted state simulated intestinal fluid; FeSSIF, fed state simulated intestinal fluid; GFR, glomerular filtration rate; Ka, acid dissociation constant; P, partition coefficient.

**Table 2:** Fitting parameters for the empirical Weibull dissolution function used in the final PK-Sim^®^ model at different doses and different studies.

	Study		
Parameter ^a^ (Unit)	1407-0001	1407-0014	1407-0002
Colon transversum solubility (mg/L)	637	637	637
Dissolution shape fasted	0.58	0.78	0.78
Dissolution shape fed			0.16
Time to reach 50% dissolution (min)			
25 mg fasted	404		243
50 mg fasted	728	442	328
100 mg fasted	1052		370
200 mg fasted	1946		736
400 mg fasted	2570		
50 mg fed			9.9
200 mg fed			293
400 mg fed			5000

^a^ Measured FaSSIF-24 h was used as reference solubility to calculate pH-dependent solubility in unmodified segments of the GI tract in a fasted state.

**Table 3:** Parameter values from the empirical Weibull function fitted to observations after administration of 50 mg in study 1407-0014.

Parameter ^a^ Unit	Value
Colon transversum solubility (mg/L)	637
Dissolution shape	0.79
Dissolution time (min)	442

^a^ Measured FaSSIF-24 h was used as a reference solubility to calculate pH-dependent solubility in unmodified segments of the GI tract in a fasted state.

**Table 4:** Observed and simulated drug–drug interaction in fasted subjects in study 1407-0014. A BI 730357 50 mg tablet was given on Day 1, with or without 200 mg itraconazole once daily from Day −3. Simulations were based on 100 virtual individuals.

		BI 730357	BI 730357 with Itraconazole	
	Parameter (Unit)	Geometric Mean (5%, 95% Percentiles)		Ratio ^a^
Observed drug–drug interaction	AUC_∞_ (nmol·h/L)	6960 (4040, 9890)	65,600 (35,600, 107,000)	9.43
	AUC_last_ (nmol·h/L)	6650 (3840, 9570)	43,200 (26,600, 60,000)	6.50
	C_max_ (nM)	186 (131, 280)	311 (218, 422)	1.67
Simulated drug–drug interaction	AUC_∞_ (nmol·h/L)	8770 (4690, 15,000)	60,800 (15,600, 142,000)	6.93
	AUC_last_ (nmol·h/L)	8590 (4650, 14,500)	40,000 (15,500, 67,300)	4.66
	C_max_ (nM)	160 (199, 215)	256 (182, 344)	1.60

^a^ Drug–drug interaction ratios were derived by dividing parameter values from BI 730357 with itraconazole treatment by parameter values from BI 730357 treatment.

**Table 5:** Simulated rifampicin drug–drug interaction based on empirical absorption after administration of 400 mg BI 730357 tablet formulation under fed conditions in studies 1470-0001 and 1407-0002.

	Simulations Using Empirical Absorption	
Parameter (Unit)	Study 1407-0001 Study 1407-0002	
AUC ratio ^a^	0.17	0.16
C_max_ ratio ^a^	0.36	0.39
AUC_∞_ (nmol·h/L)	7820	5720
C_max_ (nM)	380	511

^a^ Drug–drug interaction ratios were derived by dividing parameter values from BI 730357 with itraconazole treatment by parameter values from BI 730357 treatment.

**Table 6:** Simulated typical individual (as median of the study population) AUC and C_max_ compared with median observations based on the single-dose study data after once-daily IV solution and tablet administration in studies 1407-0001, 1407-0002, and 1407-0033.

	IV	Oral Solution		Oral Tablet			
Parameter ^a^	100 µg	2 mg	25 mg	50 mg	100 mg	200 mg	400 mg
AUC ratio	0.91	1.04	1.19	1.14	0.81	1.18	1.14
C_max_ ratio	0.74	0.60	0.79	1.09	0.95	0.93	1.00

^a^ Ratios obtained by dividing value derived from observations by value simulated using empirical absorption.

**Table 7:** Simulated AUC and C_max_ compared with observations on Day 14 after once daily tablet administration in study 1407-0002.

	Fasted State				Fed State		
Parameter ^a^	25 mg	50 mg	100 mg	200 mg	50 mg	200 mg	400 mg
AUC ratio	0.90	0.88	0.87	0.77	1.28	1.26	0.92
C_max_ ratio	0.98	1.02	0.89	0.92	1.28	1.20	1.01

^a^ Ratios obtained by dividing value derived from observations by value simulated using empirical absorption.

## Data Availability

Data cannot be shared due to commercial restrictions.

## Supplementary Materials

The following supporting information can be downloaded at: https://www.mdpi.com/article/10.3390/pharmaceutics17030314/s1, Figure S1: Exploratory plot of dose normalized plasma concentrations after oral administration of BI 730357 tablets in study 1407-0002; Table S1: Clinical data.
