# Supplementary material for: Assessing Drug–Drug Interaction and Food Effect for BCS Class 2 Compound BI 730357 (Retinoic Acid-Related Orphan Receptor Gamma Antagonist, Bevurogant) Using a Physiology-Based Pharmacokinetics Modeling (PBPK) Approach with Semi-Mechanistic Absorption
Source: Pharmaceutics. 2025 Mar 1;17(3):314. doi: 10.3390/pharmaceutics17030314 (PMC11945243; doi:10.3390/pharmaceutics17030314)
Supplement: Supplementary file 1 [file pharmaceutics-17-00314-s001.zip › pharmaceutics-3412528-supplementary.pdf]

**Table S1.** Clinical data.

| Study number[a] | Formulation, tablet milling grade | Model building | | | Model qualification | | |
|---|---|---|---|---|---|---|---|
| | | Dose group[b] | Measurement matrix | Subjects (n) | Dose group[b] | Measurement matrix | Subjects (n) |
| 1407-0001 | Oral solution | 2 mg | Plasma, urine | 6 | 8 mg | Plasma | 6 |
| | | 8 mg | Urine | 6 | | | |
| | | 25 mg | Plasma | 12 | | | |
| | Tablet, regularly milled1 | 25 mg | Plasma | 17 | | | |
| | | 50 mg | | 6 | | | |
| | | 100 mg | | 6 | | | |
| | | 200 mg | | 6 | | | |
| | | 400 mg | | 6 | | | |
| | | Fed[c], 25 mg | | 12 | | | |
| | | Fed[c], 400 mg | | 5 | | | |
| | | Fed[d], 400 mg | | 6 | | | |
| 1407-0002 | Tablet, regularly milled2 | 25 mg | Plasma | 9 | Once daily 25 mg | Plasma Day 14 | 9 |
| | | 50 mg | | 9 | Once daily 50 mg | | 9 |
| | | 100 mg | | 9 | Once daily 100 mg | | 9 |
| | | 200 mg | | 9 | Once daily 200 mg | | 9 |
| | | Fed[d], 50 mg | | 8 | Fed[d], once daily 50 mg | | 8 |
| | | Fed[d], 200 mg | | 9 | Fed[d], once daily 200 mg | | 9 |
| | | Fed[d], 400 mg | | 9 | Fed[d], once daily 400 mg | | 9 |
| 1407-0014 | Tablet, regularly milled2 | 50 mg | Plasma | 14 | 50 mg Day 1 + 200 mg itraconazole once daily from Day -3 | Plasma | 14 |
| 1407-0031 | Oral solution | 50 mg | Plasma | 6 | 50 mg | Plasma, urine | 6 |
| 1407-0033 | Intravenous radiolabelled solution | 100 mg | Plasma | 6 | | | |

[a] All studies were Phase I, enrolling healthy male subjects
[b] Single dose under fasted conditions, unless otherwise stated
[c] Standard high-fat, high-calorie meal (150 kcal protein; 250 kcal carbohydrate, 500-600 kcal fat)
[d] Standard continental breakfast (88 kcal protein; 133 kcal carbohydrate, 237 kcal fat)

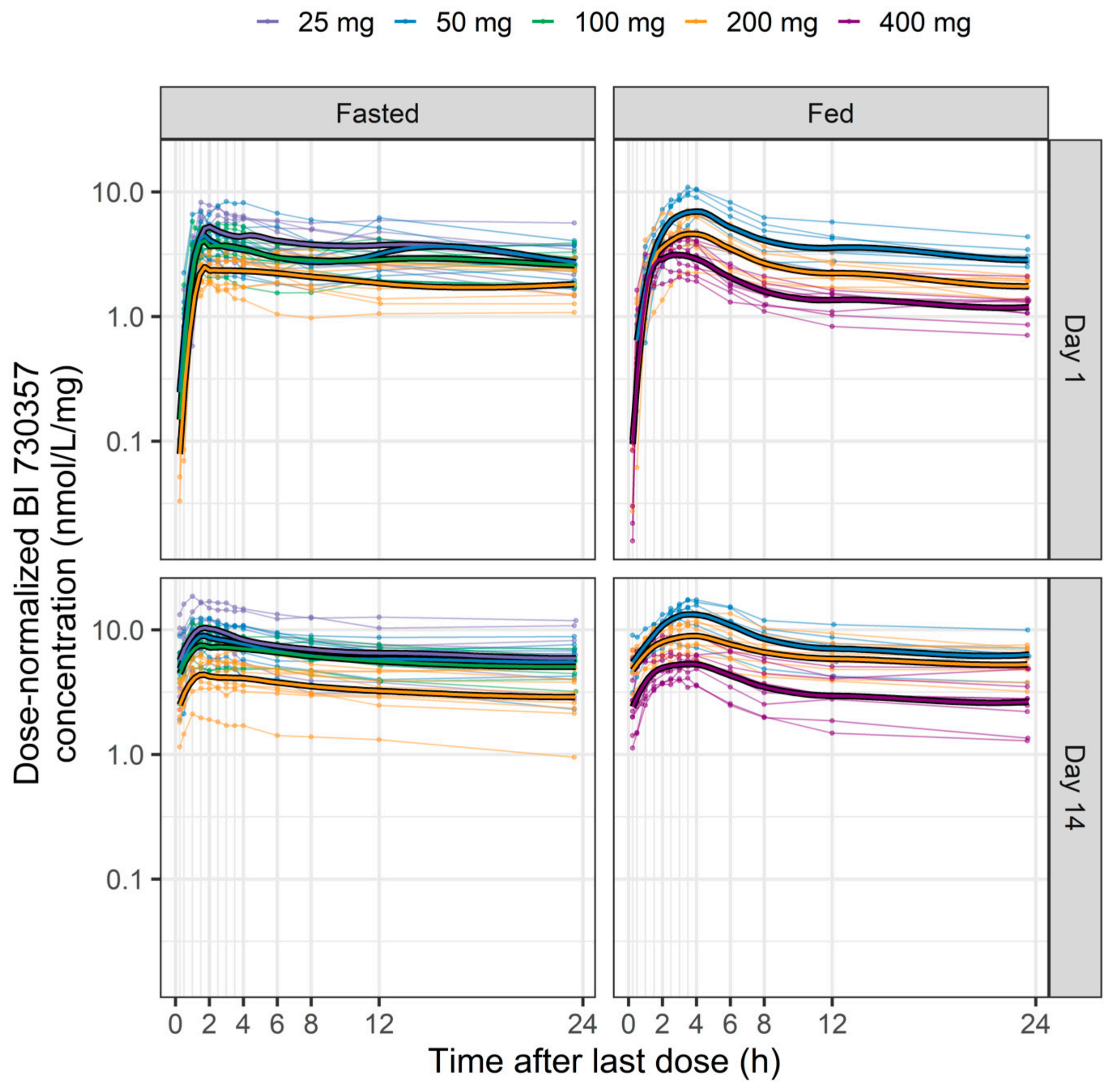


**Figure S1.** Exploratory plot of dose normalized plasma concentrations after oral administration of BI 730357 tablets in study 14070002.
